# Study of the effect of the introduction of mitochondrial import determinants into the gRNA structure on the activity of the gRNA/SpCas9 complex in vitro

**DOI:** 10.18699/VJ20.643

**Published:** 2020-08

**Authors:** E.G. Zakirova, Y.V. Vyatkin, N.A. Verechshagina, V.V. Muzyka, I.O. Mazunin, K.E. Orishchenko

**Affiliations:** Institute of Cytology and Genetics of Siberian Branch of the Russian Academy of Sciences, Novosibirsk, Russia; AcademGene Ltd., Novosibirsk, Russia; Immanuel Kant Baltic Federal University, Kaliningrad, Russia; Novosibirsk State University, Novosibirsk, Russia; Skolkovo Institute of Science and Technology, Skolkovo, Russia; Institute of Cytology and Genetics of Siberian Branch of the Russian Academy of Sciences, Novosibirsk, Russia

**Keywords:** mitochondrial DNA, CRISPR/Cas9, the mitochondrial import determinants, heteroplasmy, митохондриальная ДНК, CRISPR/Cas9, детерминанты импорта в митохондрии, гетероплазмия

## Abstract

It has long been known that defects in the structure of the mitochondrial genome can cause various neuromuscular and neurodegenerative diseases. Nevertheless, at present there is no effective method for treating mitochondrial diseases. The major problem with the treatment of such diseases is associated with mitochondrial DNA (mtDNA) heteroplasmy. It means that due to a high copy number of the mitochondrial genome, mutant copies of mtDNA coexist with wild-type molecules in the same organelle. The clinical symptoms of mitochondrial diseases and the degree of their manifestation directly depend on the number of mutant mtDNA molecules in the cell. The possible way to reduce adverse effects of the mutation is by shifting the level of heteroplasmy towards the wild-type mtDNA molecules. Using this idea, several gene therapeutic approaches based on TALE and ZF nucleases have been developed for this purpose. However, the construction of protein domains of such systems is rather long and laborious process. Meanwhile, the CRISPR/Cas9 system is fundamentally different from protein systems in that it is easy to use, highly efficiency and has a different mechanism of action. All the characteristics and capabilities of the CRISPR/Cas9 system make it a promising tool in mitochondrial genetic engineering. In this article, we demonstrate for the first time that the modification of gRNA by integration of specific mitochondrial import determinants in the gRNA scaffold does not affect the activity of the gRNA/Cas9 complex in vitro.

## Abstract (ru)

О том, что нарушения структуры митохондриального генома приводят к широкому спектру нейромышечных и нейродегенеративных заболеваний, известно уже давно, но до сих пор не найдено эффективного метода лечения болезней митохондриального происхождения. В основном проблемы с терапией подобных за- болеваний обусловлены состоянием гетероплазмии митохондриальной ДНК (мтДНК). Ввиду многокопийности митохондриального генома мутантные копии мтДНК часто сосуществуют с молекулами дикого типа в одной органелле. Клинические симптомы митохондриальных заболеваний и степень их манифестации напрямую за- висят от количества мутантных молекул мтДНК в клетке. Смещая уровень гетероплазмии в сторону молекул дикого типа мтДНК, возможно добиться снижения негативного влияния мутации. Для этой цели разработано несколько генно-терапевтических подходов на основе TALE-нуклеаз и нуклеаз типа «цинковые пальцы», однако конструирование белковых доменов таких систем является долгим и трудоемким процессом. Cистема CRISPR/ Cas9 принципиально отличается от данных систем простотой использования, высокой эффективностью и механизмом действия. Все присущие характеристики и возможности системы делают ее перспективным инстру- ментом в области генетической инженерии митохондрий. В настоящей статье мы впервые демонстрируем, что модификации направляющей РНК за счет вcтройки последовательностей, способствующих импорту нРНК в ми- тохондрии, не влияют на функциональную активность комплекса нРНК/SpCas9 в условиях in vitro. Полученные результаты указывают на возможность модификации системы с сохранением ее функциональности и использо- вания в перспективе для редактирования митохондриального генома.

## Introduction

CRISPR/Cas9 methodology is based on bacterial and archaean defense systems against viruses, transposable genetic elements, and other exogenous DNA species. Lately it has been widely utilized as an efficient multifunctional instrument for genome editing across taxonomy. Its mechanism of action differs from the one for zinc-finger nucleases (ZFNs) and TALE-nucleases (TALENs) and is based on the recognition of a target genome sequence by 20 nucleotide spacer guide RNA (gRNA) and further introduction of a double-stranded break (DSB) via recruitment of Cas9 nuclease (Jinek et al., 2012).

The first necessary step for site-specific DNA recognition and cleavage is the formation of a functional effector complex (Jinek et al., 2014; Jiang, Doudna, 2017). The identification of a target DNA sequence and the consequent nuclease conformational change proceeds via binding of hairpin loops at the 3′ end of gRNA to aminoacids of Cas9 nuclease domain, which in turn leads to the induction of nuclease activity (Wright et al., 2015). The level of complementarity between specific gRNA and Cas9 enzyme determines the thermodynamic stability of a complex and as a result the effectiveness of a target DNA cleavage (Anders et al., 2014). It was shown previously by using crystallography that four base pairs (bps) in the hairpin loops ‘tetraloop’ and ‘stem loop 2’ of a guide RNA extend beyond the ribonucleoprotein complex gRNA/Cas9 while not participating in the interaction with the side amino acid chains of Cas9 (Nishimasu et al., 2014; Konermann et al., 2015). We hypothesize that a substitution of these ‘loose’ gRNA loops with analogous hairpin structures derived from other RNA species does not affect the activity of the complex. Similar RNA modifications have been tested in studies on epigenetic regulation of nuclear gene expression (Mali et al., 2013; Konermann et al., 2015; Komor et al., 2017). It becomes obvious that gRNA molecules could be adjusted for mitochondrial genome editing as well.

A multitude of RNA species with diverse functions are expressed in eukaryotic cells. At the same time, only a minor fraction of them could be transported into mitochondria (Jeandard et al., 2019). The transport of nucleic acids into mitochondria has been a major point of a disagreement in the scientific field, therefore the application of CRISPR/Cas9 system for the suppression of mitochondrial DNA (mtDNA) mutations is generally considered questionable. However, some studies suggest the existence of specific pathways of a targeted import of cytosolic RNAs into mitochondria. Partial mitochondrial localization of synthetic RNAs modified with F- and D-domains of yeast tRNALys (CUU) has been demonstrated for yeast mitochondria undergoing stress (Martin et al., 1979; Kamenski et al., 2007). By adding similar hairpin structures others have built recombinant RNA molecules for effective import into mammalian mitochondria and consequent specific inhibition of mtDNA replication (Comte et al., 2013; Tonin et al., 2014). Similar studies utilizing RNA components of RP (Doersen et al., 1985; Holzmann et al., 2008) and MRP (Chang, Clayton, 1987) mitochondrial ribonucleases suggest that their domains can participate in targeted nucleic acid transport into these organelles (Wang et al., 2012). All the described cytosolic RNA species transported into mitochondria are short, non-coding, and contain palindromic sequences for hairpin formation, which are necessary for this type of RNA transport. Artificial introduction of such secondary structures into a gRNA could potentially facilitate RNA transition into mitochondrial matrix.

In this study for the first time, we modify RNA component of CRISPR/Cas9 complex – gRNA for its specific transport inside mitochondria. Knowing that protein component of CRISPR/Cas9 has been already adapted for mitochondrial import (Orishchenko et al., 2016), we hypothesize that reprograming of gRNAs will enable to regulate mammalian mtDNA heteroplasmy level.

## Materials and methods

**Plasmids and constructs.** A fragment of human mitochondrial DNA (DNA substrate), including protospacer in mtND1 gene, was amplified by PCR with L2797 5′-GTCCTAAACTAC CAAACCTGC-3′ and H3733 5′-ATGATGGCTAGGGTG ACTTC-3′ primers and Q5 polymerase (NEB). Guide RNAs (gRNAs) were designed for the target mtDNA sequence by online cloud-based informatics platform Benchling (https://benchling.com/). gRNA with the least number of off-target sites was chosen using the online service http://crispr.mit.edu/. Maps of the gRNA plasmids were designed in SnapGene software (https://www.snapgene.com/). All sequences of the modified gRNAs were analyzed in silico to predict secondary structure by RNAfold software from the ViennaRNA Package (Lorenz et al., 2011). To assemble plasmids for gRNA expression, oligonucleotides with overlapping ends were hybridized and inserted into the gRNA_Cloning Vector, kindly provided by Dr George Church (Addgene plasmid # 41824; http://n2t.net/addgene:41824; RRID: Addgene_41824), by Gibson assembly (NEB, USA) according to the manufacturer’s instructions. Plasmid sequences were confirmed by Sanger sequencing.

**In vitro cleavage assay.** Guide RNA was in vitro transcribed using HiScribe™T7 Quick High Yield RNA Synthesis Kit (NEB E2050) and the DNA template generated by PCR from the gRNA plasmids with primers T7_wtgRNA 5′-TAATAC GACTCACTATAGGGAGTTTTATGGtGTCAGCG-3′ and R_T7_gRNA_Cas9 5′-AAAAAAAGCACCGACTCGGT GCC-3′. RNAs were purified by phenol-chloroform extraction and ethanol precipitated. The concentration of RNA was measured using a NanoDrop 2000C spectrophotometer (Thermo Fisher Scientific) and diluted to 300 nM. Cleavage reactions were carried out in a total volume of 30 μl and contained 1 μl 1 μM nuclease Cas9 Streptococcus pyogenes (NEB M0386L) (~30 nM), 3 μl 10× reaction buffer (NEB B0386A), 1 μl gRNA (300 nM). The reaction volume was adjusted with nucleasefree water. After preliminary incubation for 10 min at 25 °C, 1 μl of 30 nM DNA substrate was added to the reaction mixture and incubated at 37 °C for 45 min. The reactions were stopped by the addition of 1 μl Proteinase K (20 mg/ ml) and incubated at room temperature for 10 min. Cleavage products were analyzed by electrophoresis on a 1.5 % agarose gel. The presence of 678 and 298 bp fragments is indicative of a specific cleavage of the DNA substrate. In vitro cleavage reactions were performed in three independent repeats. The cutting efficiency of the DNA substrate was determined by quantitative assessment of DNA in the bands by gel densitometry in Image Lab software (Bio-Rad, USA). Significant differences were calculated by Student’s t-test complex. The differences were considered significant at a significance level of p ≤ 0.05.

## Results

**Fig. 1.**
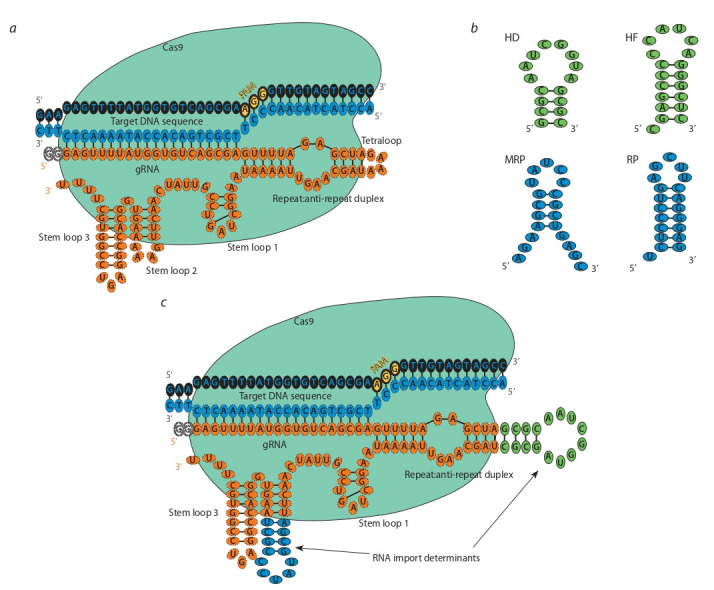
Design of recombinant gRNA. a – organization of the target DNA-gRNA-Cas9 complex, where the tetraloop and stem loop 2 of gRNA are free from the interaction with the nuclease; b – the hairpin structures are proposed to act as mitochondrial RNA import determinants; c – the substitution of a part of the gRNA scaffold loops with import determinants.

**Design of modified guide RNAs.** To study the effect of modifications in the nucleotide sequence of the guide RNA on the activity of the CRISPR/Cas9 system in vitro, their primary structure was designed. Since the tetraloop and the stem loop 2 of the constitutive part of gRNA partially extend beyond the ribonucleoprotein complex (Nishimasu et al., 2014) (Fig. 1, a), the introduction of modifications to these loci most likely should not affect the binding of the gRNA/Cas9 complex to a target DNA sequence, as well as its functional activity. Therefore, the GAAA nucleotides of the corresponding loops (tetraloop or stem loop 2) of the gRNA were replaced by a nucleotide sequence of one of the four mitochondrial RNA import determinants (HD, HF, RP, MRP hairpins) (see Fig. 1, b) in all possible conformations (direct, reverse, complement, reverse-complement). We designed 32 variants of gRNA with insertion of mitochondrial RNA import determinants in different conformations into the tetraloop or the stem loop 2. For each variant of the modified gRNA, a secondary structure was predicted using RNAfold web server from the ViennaRNA software package (Lorenz et al., 2011). The in silico predicted structures of the modified gRNAs were compared with the theoretical one for non-modified gRNA. Eventually for each of the import determinants inserted in the tetraloop or the stem loop 2, one of the most optimal conformation was selected. A total of eight variants of modified gRNA were obtained; secondary structures of which had minimal differences from unmodified gRNA (see the Table). All variants of modified gRNA were cloned into a gRNA-cloning vector. The HF-SL variant has not be cloned due to technical difficulties, which are most likely associated with the secondary structure in the nucleotide sequence.

**Table 1.**
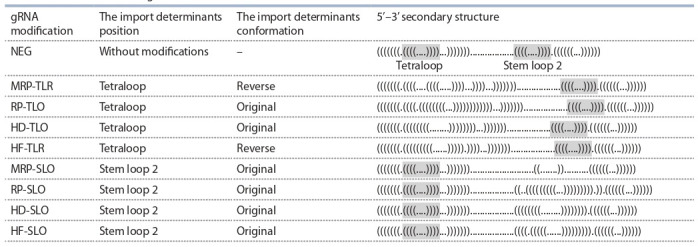
Characteristics of modified gRNAs Note. The secondary structure of gRNAs is represented as dot-bracket notation. Each symbol corresponds to a base in the gRNA. The bracket denotes a paired base pair located in the sequence. The dots denote unpaired bases which correspond to loops in the hairpin structure. Unmodified tetraloop and stem loop 2 highlighted in gray.

**Fig. 2.**
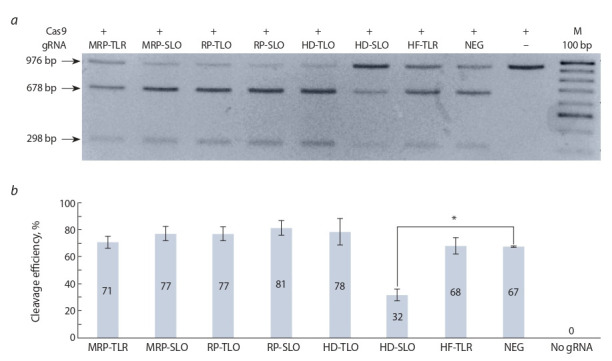
In vitro DNA cleavage by the gRNA/SpCas9 complex with modified gRNA. Cleavage efficiency was assessed by agarose gel electrophoresis (a) and measured using densitometry (b). Standard deviation from the mean is shown as error bars (+/–). M – 100 bp DNA ladder. Statistical assessment is made by Student’s t-test; * indicate significant differences between NEG and modified gRNA, p ≤ 0.05.

**Analysis of the effect of gRNA modifications on the functional activity of the gRNA/Cas9 complex in vitro.** The activity of the Cas9 nuclease in a complex with modified gRNA was assessed using in vitro cleavage reactions. The reactions used gRNA synthesized by in vitro transcription with T7 RNA polymerase and recombinant Cas9 nuclease from S. pyogenes. A double-stranded DNA fragment of 976 bp amplified by PCR was used as a substrate for in vitro cleavage reactions. The protospacer was selected to form two fragments of 298 and 678 bp long, if a double-stranded break in the DNA substrate was successfully introduced by the gRNA/Cas9 complex. Control in vitro cleavage reactions were performed with unmodified gRNA (NEG) and without the addition of any gRNA. All reactions were carried out in three independent repeats. The results of agarose gel electrophoresis of the products of in vitro cleavage reactions are presented in Fig. 2, a. As shown in Fig. 2, a, using all variants of modified gRNAs, a specific cleavage of the DNA substrate occurs and the fragments of the expected size are formed. Thus, despite the modifications in the structure of the gRNA, the gRNA/ Cas9 complex retains its activity.

A quantitative analysis of the efficiency of the DNA substrate cutting was carried out using densitometry. The cleavage efficiency was determined by the ratio of the pixel density in the bands corresponding to the cleaved DNA substrate to the original uncut DNA fragment (see Fig. 2, b). The efficiency of cutting the DNA substrate with Cas9 nuclease in complex with unmodified gRNA (NEG) is 67 %. Modification of gRNA by inserting the HD hairpin in direct conformation into the stem loop 2 of gRNA (HD-SLO variant) significantly ( p ≤ 0.05) reduced the efficiency of DNA substrate cleavage to 32 %, i. e. more than twice compared to unmodified gRNA (NEG). Other variants of gRNA modifications did not lead to any statistically significant changes in the efficiency of DNA substrate cleavage.

## Discussion

CRISPR/Cas9 methodology is a revolutionary approach for nuclear genome editing. It has broadened our capabilities for the basic studies of biological processes as well as in the development of human disease therapies. CRISPR/Cas9 adaptation for mtDNA editing is an exciting topic for many laboratories worldwide (Verechshagina et al., 2019). However, unambiguous demonstration of its effective functioning in mitochondria remained unresolved supporting the current opinion about impracticality of using CRISPR/Cas9-derived systems for mitochondrial genome manipulation (Gammage et al., 2018).

Various complications with adaptation of this system for mitochondria are associated with inaccessibility of mitochondrial matrix for the system components due to the presence of outer and inner mitochondrial membranes. A mitochondrion consists of approximately 1500 different proteins with diverse functions, and only 13 of them are encoded by mtDNA and synthetized in the organelle itself (Calvo, Mootha, 2010). Unsurprisingly, there are many known mechanisms of protein transport into different mitochondrial subcompartments (Pfanner et al., 2019). We (Orishchenko et al., 2016) and others (Jo et al., 2015; Loutre et al., 2018; Bian et al., 2019) have demonstrated that introduction of a mitochondrial localization signal at the N-terminus of Cas9 leads to an effective Cas9 import into mitochondrial matrix. Therefore, protein component of CRISPR/Cas9 system could be imported inside mitochondria.

The second task in CRISPR/Cas9 adaptation for mtDNA modification is gRNA mitochondrial import. Unfortunately, the molecular mechanisms of RNA transport across mitochondrial membranes have not been unambiguously described yet. Moreover, there is no consensus on RNA species imported, their function in mitochondria, and intramembrane channels through which they get transferred. Therefore, development and optimization of gRNA mitochondrial import represents a bottleneck in the overall adaptation of the system

On the contrary, there have been many recent publications demonstrating successful import of diverse RNA species into mitochondria (Rubio et al., 2008; Wang et al., 2010; Fan et al., 2019; Jeandard et al., 2019). Generally, import of these RNAs is mediated by a stem-loop type hairpin structures. It has been shown that HF and HD hairpins in yeast Saccharomyces cerevisiae tRNA are responsible for intramitochondrial transport of tRNA^Lys^ CUU (tRK1). Introduction of these hairpins to other RNAs leads to their in vivo mitochondrial import with a consequent restoration of functions initially disturbed by mtDNA mutations (Kazakova et al., 1999; Kamenski et al., 2010; Gowher et al., 2013; Tonin et al., 2014). Additionally, it was demonstrated that RP and MRP hairpins mediate mitochondrial import of H1 and 7-2 RNAs respectively (Wang et al., 2010; Noh et al., 2016; Markantone et al., 2018) thus suggesting that the addition of such components into gRNA structure enables their effective transfer into mitochondrial matrix.

In earlier studies both components of CRISPR/Cas9 system have been extensively modified to achieve high effectiveness and specificity, and to increase its potential functional repertoire. There are several main structural elements of a gRNA: a spacer – a sequence approximately 20 nt in length at the 5′ end of gRNA which is complementary to a target genome sequence, and four hairpins – secondary RNA structures of a stem-loop type (tetraloop, stem loop 1/nexus, stem loop 2, and stem loop 3). The tetraloop contains a lower stem, an overhang, and an upper stem (Briner et al., 2014; Nishimasu et al., 2014). By using site-directed mutagenesis it was shown that the overhang and the stem loop 1/nexus are the key elements of a gRNA necessary for the action of the CRISPR/Cas9 complex. At the same time, the upper stem in the tetraloop and the stem loop 2 could be substantially modified or even eliminated from the gRNA while not compromising gRNA/ Cas9 complex activity (Briner et al., 2014; Konermann et al., 2015). Moreover, lengthening of the tetraloop and the stem loop 2 increases the stability of the gRNA and the effectiveness of an assembly of the complex gRNA/dCas9 (Ma et al., 2016; Shao et al., 2016). Therefore, we hypothesized that introducing mitochondrial localization signals into these hairpin structures will facilitate intramitochondrial transfer of gRNAs without affecting the functional performance of gRNA/Cas9 complex.

In the current study, we add HD, HF, RP, and MRP hairpins in different conformations into the tetraloop or the stem loop 2 gRNA structures. Subsequently, using in vitro cleavage assay we assess the effects of gRNA modifications on the activity of gRNA/Cas9 complex. We detect specific cleavage of DNA substrates by the combinations of Cas9 with every of our modified gRNA variants (see Fig. 2, a) which suggests that introduced modifications do not affect the formation of gRNA/Cas9 complex, as well as the specificity of DNA binding. Importantly, some of gRNA modifications lead to both the increase and the decrease of DNA substrate cleavage rate which could be associated with the influence of the modifications on the gRNA stability, the effectiveness of gRNA/Cas9 complex formation, and Cas9 nuclease activity (Nowak et al., 2016).

An analogous approach for gRNA transport into mitochondrial matrix was taken by R. Loutre and colleagues even though the import determinants were added at either the 5′ or the 3′ end of the gRNA (Loutre et al., 2018). In the case of the 3′ end modification, the activity of gRNA/Cas9 complex in vitro matches its activity while using unmodified gRNA. On the contrary, the 5′ end modification significantly diminishes the complex activity. Most likely, this effect is associated with processing and degradation of the 5′ end of modified gRNA. This has been demonstrated for gRNAs with an increased spacer region as well as with an insertion of MS2 and PP7 hairpins at the 5′ end (Ran et al., 2013; Zalatan et al., 2015; Nowak et al., 2016). The 3′ end modifications could decrease the expression and the stability of a gRNA which affects the activity level of gRNA/Cas9 complex (Zalatan et al., 2015).

## Conclusion

Therefore, the tetraloop and the stem loop 2 are potentially the optimal regions for the insertion of mitochondrial localization sequences. However, these variants should be tested not only in vitro but in vivo in cell cultures to analyze both gRNA/Cas9 complex activity and the effectiveness of intramitochondrial transport of both components.

## Conflict of interest

The authors declare no conflict of interest.
